# Day 5 vitrified blastocyst transfer versus day 6 vitrified blastocyst transfer in oocyte donation program

**DOI:** 10.1038/s41598-021-90238-y

**Published:** 2021-05-21

**Authors:** G. M. Yerushalmi, T. Shavit, S. Avraham, M. Youngster, A. Kedem, I. Gat, U. S. Dorofeyeva, S. Mashiach, E. Schiff, A. Shulman, D. S. Seidman, A. Wiser, E. Maman, A. Hourvitz, M. Baum

**Affiliations:** 1 IVF Unit, Department of Obstetrics and Gynecology, Shamir Medical Center, Affiliated with the Sackler Faculty of Medicine, Tel Aviv University, Tel Aviv, Israel; 2 IVF Unit, Assuta Medical Center, Tel Aviv, Israel; 3 IVF Unit, Herzliya Medical Center, Herzliya, Israel; 4 IVF Unit, Meir Medical Center, Affiliated with the Sackler School of Medicine, Tel Aviv University, Tel Aviv, Israel; 5 Medical Center Interserono Medicover, Lviv National Medical University, Lviv, Ukraine

**Keywords:** Embryology, Infertility

## Abstract

The superiority of day 5 blastocysts compared to day 6 blastocysts in fresh cycle transfers was previously demonstrated and attributed mainly to endometrial asynchrony. Data from frozen blastocysts transfers showed conflicting results, possibly due to heterogeneous patient population and embryo quality. The aim of this study was to compare clinical pregnancy rate (CPR) and live birth rate (LBR) between transfers of vitrified day 5 blastocysts and day 6 blastocysts in oocyte donation, blastocyst-only cycles. In a retrospective, multi-center study, with a single oocyte donation program, a total of 1840 frozen embryo transfers (FET’s) were analyzed, including 1180 day 5 blastocysts and 660 day 6 blastocysts transfers. Day 5 blastocyst transfers had better embryonic development and significantly higher CPRs (34.24% vs. 20.15%, *P* < 0.0001), higher LBRs (26.89% vs. 14.77%, *P* < 0.0001), less cycles to LBR (1.83 ± 0.08 vs. 2.39 ± 0.18, *P* = 0.003) and shorter time to LBRs (76.32 ± 8.7 vs. 123.24 ± 19.1 days, *P* = 0.01), compared to day 6 transfers, respectively. A multivariate stepwise logistic regression indicated, that day 5 transfer was an independent factor for CPRs (OR 1.91; 95% CI 1.43–2.54, *P* < 0.001) and LBRs (OR 2.26; 95% CI 1.19–4.28, *P* = 0.01), regardless of embryo quality, compared to day 6. In conclusion, day 5 blastocysts in oocyte donation program have significantly higher CPRs and LBRs, and present shorter time to delivery, compared to day 6 blastocysts, regardless of embryo quality.

## Introduction

The selection of the best embryo to transfer during elective single embryo transfer (e-SET) is a major decision in the effort to reduce the rate of multiple pregnancies, while preserving live birth rate (LBR)^1^. Improvements in culture media and vitrification methods allowed the transfer of blastocyst stage embryos in an attempt for better selection of viable embryos^2^. The debate regarding the effect of the time to reach blastocyst stage on IVF outcomes has been the focus of several studies during the last decade, but a final conclusion has not been reached. While most studies on fresh cycles showed an advantage to embryos transferred on day 5 compared to day 6^3,4^, studies on frozen embryos showed conflicting results.

The day 5 priority during fresh cycles can be explained by endometrial asynchrony or embryo quality. In frozen embryo transfers (FET’s) the endometrial factor is supposedly eliminated. A meta-analysis from 2010 demonstrated that day 6 embryos that were at the same stage of development at cryopreservation as day 5 cryopreserved embryos, had similar clinical pregnancy and live birth rates^5^. A Recent meta-analysis found that day 5 blastocysts transfers have higher clinical pregnancy (CPR) and live birth rates (LBR) compared with day 6 embryos in both frozen and fresh cycles, regardless to embryo quality, in agreement with preceding retrospective studies^6–8^. The studies included were composed of heterogeneous patients, protocols and freezing methods. Most of them were not designed to answer the main outcome of the meta-analysis^8^.

In this study, we aimed to compare clinical outcomes of day 5 and day 6 vitrified embryos. In order to control for as many confounders as we can, we analyzed FETs of blastocyst only cycles, coming from a single oocyte donation medical center.

## Materials and methods

### Study design

The study included all oocyte donation blastocyst-only transfers in 3 medical centers during the years 2015–2019. All egg donations (ED) were obtained from a single medical center in Lviv, Ukraine. Blastocysts were vitrified and shipped to the 3 medical centers where transfers were done. The study was approved by the local institutional review board (IRB) in each of the 3 centers (Shamir, Assuta and Herzliya medical centers). Informed consent was waved by the IRB. Day 5 blastocyst transfers were retrospectively compared to day 6 blastocyst transfers. We also compared day 5 and day 6 FETs in the first FET cycle of each patient.

All methods were performed in accordance with the guidelines regarding clinical retrospective studies.

### Patients

All oocyte recipients in 3 medical centers during the time study were included. We included blastocyst only cycles. We excluded two embryo mixed day 5 and day 6 FETs. Endometrium preparation was programmed with hormone replacement therapy (HRT) or based on the natural cycle (NC), according to the physician's preference. All FETs were performed after 5 days of progesterone exposure, 2–4 h after thawing. The transfers were only with frozen embryos, due to cross border embryo transport. No fresh ED embryo transfers are routinely performed in the medical centers that participated in the study.

### Donors

All egg donations were from a single medical center in Lviv, Ukraine. Donors were anonymous, 20–32 old, with appropriate ovarian reserve tests (AMH > 2, AFC > 16). Each donor was allocated to a maximum of two recipients. The donors underwent a uniform GnRH antagonist stimulation protocol with recombinant gonadotropins and GnRH agonist triggering. Fresh oocytes were fertilized. Fertilization was undertaken in Lviv with shipped partners’ or donors’ sperm using ICSI. Sperm was defined according to its concentration: > 1 million/ml (1) or lower (0) in order to avoid a possible difference due to of poor sperm quality on embryonic outcome. All embryos were cultured and vitrified on day 5 or day 6 according to the embryologist decision and by their stage of development. This was done by one of four senior embryologists. Only good quality blastocysts (3BB or higher grade) were included. Blastocysts were vitrified using the SAGE® Vitrification Kit according to the manufacturer specifications. Frozen embryos were shipped to the 3 medical centers and warmed before transfer with the SAGE® Vitrification warming Kit.

### Embryos and blastocysts grading

Day 3 embryos were graded based on the following morphological criteria: Top—8 blastomeres, < 5% fragmentation, Good——6–8 blastomeres, < 10% fragmentation and Fair—< 6 blastomeres, > 10% fragmentation.

For the purpose of defining weighted score for day 3 embryos, top embryos received 3 points, good received 2 points and fair embryos 1 point.

Blastocysts were scored according to their expansion, inner cell mass (ICM) and trophectoderm (TE) development: 1—Early, 2—Blastocyst, 3—Expanded, 4—Hatched. **ICM**: A—Prominent, easily discernible, with many cells that are compacted and tightly adhered together, B: Easily discernible, with many cells that are loosely grouped together, C: Difficult to discern, with few cells. **TE** A: Many cells forming a cohesive epithelium, B: Few cells forming a loose epithelium, C: Very few cells.

The analysis and comparison between day 5 and 6 blastocysts was based on classification of 3 groups: Top blastocysts (4AA), good (3,4 AB, BA), and fair (BB). Lower quality blastocysts were not frozen or transferred.

### General characteristics and outcomes measured

We recorded cycle baseline characteristics and embryonic development parameters for each transfer performed: age of recipient on transfer, age of the donor during stimulation, number of oocytes allocated for the patient, fertilization rate (per M2 oocyte), cleavage rate (per M2 oocyte), weighted day 3 score, blastocyst development rate (day 5 BB and above per 2PN)^9^, freezing rate (blastocysts frozen per M2), and embryo quality at transfer.

The main outcomes measured were CPR and LBR. CPR was defined as pregnancy with fetal heart beat observed in first trimester ultrasound. LBR was calculated as births out of embryo transfers before February 2019, since 25% of the pregnancies were ongoing at the time of statistical analysis. Missed abortion (MA) rate was defined as loss of fetal heart rate before 20 weeks of pregnancy. Main outcomes were elaborated also according to the 3 groups of embryo quality.

We examined all the cycles that led to delivery and calculated for them the time to pregnancy in days (date of FET minus date of first FET) and total cycle number. Although multiple factors could affect this time (patient's time-off etc.), the calculation was meant to represent real-life time to pregnancy.

Due to the possible bias of preferring transfers of day 5 blastocysts on the first FET we compared the first transfer of two groups of patients: Patients that had day 5 embryos only or patients with day 6 embryos only.

### Statistical methods

The groups were compared using Pearson’s Chi-square test or Fisher’s exact test for dichotomous variables and Student’s T-test for normally distributed continuous variables and Mann Whitney test for continuous variables not normally distributed. A *P* value < 0.05 was considered significant.

In order to identify the effect of day 5 embryos vs day 6 embryos adjusted to potential confounding factors that could be independently associated with CPR and LBR, we performed a multiple variables stepwise logistic regression model, with the following parameters included: day 5 or 6, recipient age, donor age, sperm quality, embryo quality at transfer, weighted quality score at day 3, freezing rate, cleavage rate, blastulation rate and total cycles to pregnancy.

Also, since some patients had more than one transfer, a GEE model with independent correlation matrix was applied. The number of cycles per patient was not related to clinical pregnancy and live birth and did not change the analysis results.

## Results

During the study period a total of 1898 FETs to 783 patients were performed. The oocytes were obtained from 517 donors. Fifty-eight cycles were omitted from the analysis including 22 FETs that consisted of double simultaneous transfers of day 5 and day 6 blastocysts and 36 cycles that missed data crucial for the analysis.

Hence, analysis was carried out for 1840 blastocyst transfers, 1180 day 5 embryos FET’s and 660 day 6 embryos FETs. LBR was calculated for 1389 ET’s, 915 day 5 transfers and 474 day 6 transfers. Overall CPR was 29.18% with 22.75% live birth rate and 9.12% miscarriage rate.

Day 5 embryos FETs compared with day 6 embryos FETs had similar donor age, recipient age, sperm quality and mean number of oocytes received per patient. Significant difference was demonstrated in the fertilization (85.12% vs. 83.62%, *P* = 0.007) and cleavage rates (83.79% vs. 81.83%, *P* = 0.0008) between day 5 and day 6 FETs, respectively. The original cohort of day 5 compared with day 6 blastocyst transfers displayed significantly higher mean weighted day 3 score with significantly higher blastulation rate, freezing and survival rate. Data is presented in Table 1.

**Table 1:** Baseline and embryological characteristics of day 5 blastocysts vs. day 6 blastocysts cycles.

	Day 5 (N = 1180)	Day 6 (N = 660)	*P* value
Mean donor age (years)	26.72 ± 0.41	26.99 ± 0.12	0.08
Mean patient age (years)	43.71 ± 0.12	44.08 ± 0.18	0.09
Sperm > 1mil/ml (%)	1052/1180 (89.15)	577/660 (87.5)	0.28
Mean No. of oocytes received per patient	10.03 ± 0.14	9.94 ± 0.08	0.38
Fertilization rate (%)	10,076/11,837 (85.12)	5479/6552 (83.62)	0.007
Cleavage rate (%)	9918/11,837 (83.79)	5362/6552 (81.83)	0.0008
Weighted D3 score	2.10 ± 0.01	2.02 ± 0.01	< 0.0001
Blastocyst development rate (%)	3730/10,076 (37.02)	676/5479 (12.34)	< 0.0001
Freezing rate (%)	5402/11,837 (45.64)	2479/6552 (37.84)	< 0.0001
Survival rate (%)	1265/1333 (94.89)	693/765 (90.59)	0.0002
Transfer of 2 blastocysts (%)	88/1180 (7.46)	45/659 (6.83)	0.64

Data is presented in mean ± standard error or percentage.

Day 5 blastocysts displayed significantly higher CPR (34.24% vs. 20.15%, *P* < 0.0001) and LBR (26.89% vs. 14.77%, *P* < 0.0001) compared to day 6 blastocyst transfers. The abortion rate was higher among day 6 transfers, but the difference was marginally significant (13.53% vs. 7.67%, *P* = 0.05).

When transfers were compared according to the quality of blastocysts, day 5 blastocysts kept displaying higher CPR and LBR, but the difference reached significant difference only in the good quality group, for both outcomes, and also in the fair group for LBR. Similar results were demonstrated in regard to ongoing pregnancy rate (data not shown). Interestingly, when we compared the **fair** day 5 embryo quality group to the **good and top** day 6 embryo quality groups, day 5 embryos still exhibited significantly higher LBR (24.12% vs. 16.5%, *P* = 0.04). The mean number of cycles to live birth (1.83 ± 0.08 vs. 2.39 ± 0.18, *P* = 0.003) and mean days to pregnancy leading to live birth (76.32 ± 8.7 vs. 123.24 ± 19.1, *P* = 0.01) were higher among day 6 blastocysts compared to day 5 blastocysts transfers. Data is presented in Table 2.

**Table 2:** Clinical outcomes of day 5 blastocysts vs. day 6 blastocysts ET’s, All.

	Day 5 (N = 1180)	Day 6 (N = 660)	*P* value
**CPR (%)**			
Total	404/1180(34.24)	133/660 (20.15)	< 0.0001
Top	83/229 (36.24)	32/116 (27.59)	0.11
Good	270/753 (35.86)	72/385 (18.7)	0.0001
Fair	51/198 (25.75)	29/159 (18.23)	0.09
LBR (%) *			
Total	246/915 (26.89)	70/474 (14.77)	< 0.0001
Top	56/199 (28.14)	16/83 (19.28)	0.13
Good	149/546 (27.29)	42/267 (15.73)	0.0002
Fair	41/170 (24.12)	12/124 (9.68)	0.0019

Data is presented in mean ± standard error or percentage.

CPR—clinical pregnancy rate, *LBR—live birth rate, derived from ET before 2/2019.

Table 3 presents results of the first FET for each patient, thus minimizing the bias of preferring day 5 embryos transfers for patients with both blastocysts types available for transfer. We compared 553 day 5 blastocysts with 232 day 6 blastocysts, transferred in the first FET of each patient in the study. The results preserved the trend for better outcomes for day 5 compared with day 6 FETs.

**Table 3:** Clinical outcomes of day 5 vs. day 6 ET, First ET Only.

	Day 5 (N = 553)	Day 6 (N = 232)	*P* value
**CPR (%)**			
Total	210/553 (37.97)	52/232 (22.41)	0.0001
Top	48/116 (41.37)	19/63 (30.15)	0.11
Good	140/359 (38.99)	27/132 (20.45)	< 0.0001
Fair	22/78 (28.20)	6/37 (16.21)	0.24
LBR (%) *			
Total	135/438 (30.82)	22/167 (13.17)	0.0001
Top	32/101 (31.68)	8/39 (20.51)	0.21
Good	84/268 (31.34)	13/96 (13.54)	0.0007
Fair	19/50 (27.54)	1/31 (3.13)	0.003

Data is presented in mean ± standard error or percentage.

CPR—clinical pregnancy rate, *LBR—live birth rate, derived from ET before 2/2019.

In a multivariate logistic regression model for CPR, we found that day 5 blastocyst transfer compared to day 6 blastocyst transfer has significantly increased odds by 1.91 (95% CI 1.43–2.54). Other factors that were independently associated with clinical pregnancy included the age of the recipient, embryo quality at transfer, day of transfer and the number of cycles to pregnancy. Increased recipient age reduced the odds for clinical pregnancy by 4% (OR 0.96; 95% CI 0.94–0.99). Top quality as compared to fair quality significantly increased the likelihood for clinical pregnancy by 1.57 (95% CI 1.10–2.24), while good quality as compared to fair quality had significantly increased odds by 1.39 (95% CI 1.04–1.87).

The number of cycles to pregnancy decreased the odds for clinical pregnancy by 11% (OR 0.89; 95% CI 0.83–0.96). Data is presented in Table 4.

**Table 4:** Logistic regression model of the possible factors associated with clinical pregnancy in vitrified blastocyst cycles on egg donation program, All.

	Odds ratio	95% CI	*P* value
**Patient age**	**0.96**	**0.94–0.99**	**0.005**
Donor age	0.99	0.95–1.02	0.65
Sperm quality	1.13	0.81–1.58	0.46
**Top vs. fair**	**1.57**	**1.10–2.24**	**0.01**
**Good vs. fair**	**1.39**	**1.04–1.87**	**0.02**
Fertilization rate	0.71	0.32–1.58	0.41
Cleavage rate	0.91	0.32–2.62	0.87
Weighted score day 3	0.94	0.68–1.30	0.74
Blastulation rate	0.93	0.44–1.95	0.86
Freezing rate	1.00	0.99–1.01	0.14
**Day 5 vs. day 6**	**1.91**	**1.43–2.54**	**< 0.001**
**Total cycles**	**0.89**	**0.83–0.96**	**< 0.001**

Analysis for factors associated with LBR demonstrated that day 5 blastocysts transfers were 2.26 more likely to reach delivery compared to day 6 blastocysts transfers (95% CI 1.19–4.28) and that the number of cycles to pregnancy decreased the odds for clinical pregnancy by 12% (OR 0.88; 95% CI 0.80–0.96) (Table 5).

**Table 5:** Logistic regression model of the possible factors associated with live birth rate in vitrified blastocyst cycles on egg donation program, All.

	OR	95% CI	*P* value
**Patient age**	**0.97**	**0.94–1.00**	**0.05**
Donor age	0.98	0.94–1.02	0.34
Sperm quality	1.15	0.77–1.72	0.48
Top vs. fair	1.48	0.97–2.26	0.06
Good vs. fair	1.35	0.94–1.92	0.09
Fertilization rate	0.75	0.28–2.06	0.59
Cleavage rate	0.87	0.23–3.35	0.84
Weighted score day 3	1.27	0.85–1.89	0.23
Blastulation rate	0.7	0.23–2.1	0.52
Freezing rate	1.00	0.99–1.01	0.18
**Day 5 vs. day 6**	**2.26**	**1.19–4.28**	**0.01**
**Total cycles**	**0.88**	**0.80–0.96**	**< 0.001**

## Discussion

In this study we compared the clinical outcome of transfers of day 5 vitrified blastocysts versus day 6 vitrified blastocysts, in an oocyte donation program. We found that day 5 FETs demonstrated significantly better clinical outcomes compared to day 6 transfers, with higher CPR and LBR. The superiority of day 5 transfer was demonstrated in a univariate analysis as well as in a multiple variable stepwise logistic regression model. The time to reach pregnancy leading to live birth was also significantly longer with transfers of day 6 embryos, requiring more cycles.

We also found that although there was no difference in the demographics and basic parameters of the fresh cycle (i.e., donor age, sperm quality, number of received oocytes), day 6 embryos originated from oocytes with significantly lower fertilization rate, lower cleavage rate, lower quality day 3 embryos, lower blastulation rate, lower freezing rate and lower survival rate, compared to day 5 blastocysts. Altogether, suggesting that better quality oocytes might result in more days 5 blastocyst. Those blastocysts represent a better developmental pattern, resulting in better clinical results.

This study is the first large study examining oocyte donation vitrified blastocysts-only FETs. All blastocysts were received from a single medical center. This unique set of FETs, allowed us to analyze a large group of embryos that originated from young donors with no infertility issues and thus, have a relatively high chances of providing the best quality, with minimal heterogeneity of embryos to transfer.

Our results are in concordance with recent studies on autologous frozen blastocysts transfers. Tubbing et al. (10), showed in a small retrospective study adjusted odds ratio for clinical pregnancies and deliveries for day 5 compared with day 6 of 2.83 and 2.94, respectively. The comparison in that study was based on stage of development and not on grading, and patients were older, although adjustment for confounding factors was noted^10^. Similar to Ferruex et al. (7), we showed higher CPR’s and LBR’s in day 5 FETs that were compared with day 6. In that study, even though ages were similar between the transfer groups, older women were included. Our results confirm that transfers of day 5 blastocysts result in better clinical outcome regardless to the stage or grading of the blastocysts. Day 5 FETs consistently displayed higher pregnancy rates among all quality groups. The lack of statistical significance in the univariate analysis in some of the groups might be due to the smaller subgroup size or a limited difference when one transfers extremely excellent or less favorable embryos. Yet, when we compared the least favorable fair quality day 5 embryo group to the top and good quality day 6 embryos, day 5 embryos had a significantly better chance to achieve LBR, probably due to the larger group sizes, allowing achievement of statistical significance. Our regression models exhibit better outcomes of day 5 embryos regardless to embryo quality. It is important to notice that since no low-quality embryos were frozen or transferred, our groups were comprised of only good-quality embryos, in all groups, in contrast to previous studies. No embryos graded less than BB were included in the cohort, while these embryos were included in the “good” or “intermediate” groups in previous studies^7,10^.

Contrary to our results, El-Toukhy et al., found no difference in day 5 or day 6 transfers with 29% LBR for both groups^11^. Similar to our study, only BB graded or better blastocysts were frozen. Yet, there was a significant difference in the baseline characteristics of the patients, with better prognosis patients in the day 5 transfers group.

The difference between our results and El-Toukhy results could be explained by different cryopreservation protocol (slow freeze vs. vitrification), although that would not explain the lack of difference between 2 study groups in his study. Also noted is that in that study less than half of the cycles were single blastocyst transfer, a fact that might have compensated for lower quality embryos. In our study more than one embryo was transferred in only 7% of the cycles for both groups.

Yang et al. stated no difference in “high quality” embryos between day 5 and day 6 transfers^12^. In fact, the high quality defined by Yang was the entire study group in our research that was further sub-divided. Thus, when results are compared, we must remember that group definitions differ significantly between studies.

The multivariate logistic regression also demonstrated that younger age of the oocyte recipient was significantly associated with higher CPR and LBR. This finding is in concordance with previous studies that demonstrated higher implantation and pregnancy rates in younger recipients^13–16^, although a more recent study found no association between the recipient age and cumulative LBR^17^. The average age of recipients in that study was 41 years compared to over 43 in our study, which might explain the different results. A study that analyzed 27,959 fresh oocyte donation cycles^18^ found, that only after the age of 45, the recipients experienced statistically significant lower CPR and LBR. The reduction in our study in CPR and LBR was 4% and 3% for each year of age in the recipients, respectively. This reduction could be explained by lower endometrial receptivity with increasing age, older paternal age or obstetrical complications. Our data was missing these factors, although advanced paternal age was not shown to significantly affect fertility^19^.

Our study has several limitations. First, the retrospective nature of the study might have limited our ability to control for confounding factors. Yet, due to the large number of FETs and the use of a multivariate regression model we can assume similar distribution and control of confounding factors. We also did not have pre-implantation genetic screening performed. While some studies have found relation of chromosomal abnormalities to slower developing embryos, others found it related to morphology (which was controlled in our study), and others found no difference in ploidy rate between day 5 and day 6 blastocysts^12,20,21^. We also could not control for physician on transfer and the preparation of endometrium before transfer. Gathering data from 3 centers allowed large number of transfers to be analyzed, yet involved several physicians working. NC or HRT were selected by physician preference. We believe that the large number of transfers compensated for the possible difference between groups. Moreover, none of the preparation methods was proven to be superior in most studies^22–24^. We also had no further elaborated data on sperm counts, morphology or motility, besides mention of sperm that was lower than 1 million per ml. Although the distribution of poor sperm was similar between both groups, other parameters might have affected blastocysts development. In addition, in minority of the cases transfer was not of a single embryo. Yet, it was equal between the two groups. Also, we followed a strict freezing protocol, which included the freezing of 3BB and above graded blastocyst. Thus, our results might not be generalized to centers that freeze lower graded blastocysts on day 5.

Finally, the study involved oocyte recipients which are naturally older than the average infertility population. Thus, the results might not be extrapolated to a younger population.

In conclusion, we found that transfers of day 5 blastocysts resulted in significantly better clinical outcomes compared to transfers of day 6 blastocysts in oocyte donation blastocysts-only cycles. Day 5 embryos display better embryologic development from fertilization to blastulation, and have higher rates of pregnancy and live birth in a shorter time. This information could assist in better embryo selection in e-SET, and shorten time to pregnancy when possible.
